# Antibiotic exposure elicits the emergence of colistin- and carbapenem-resistant *Escherichia coli* coharboring MCR-1 and NDM-5 in a patient

**DOI:** 10.1080/21505594.2018.1486140

**Published:** 2018-07-26

**Authors:** Jinpeng Mao, Wugao Liu, Wei Wang, Jian Sun, Sheng Lei, Youjun Feng

**Affiliations:** a Department of Neurosurgery, Tianmen First People’s Hospital, Tianmen, China; b Clinical Laboratory of Lishui People’s Hospital, Lishui, China;; c National Risk Assessment Laboratory for Antimicrobial Resistance of Animal Original Bacteria, South China Agricultural University, Guangzhou, China; d Department of Medical Microbiology & Parasitology and Department of General Intensive Care Unit of the Second Affiliated Hospital, Zhejiang University School of Medicine, Hangzhou, China;; e College of Animal Sciences, Zhejiang University, Hangzhou, China

**Keywords:** Antibiotic exposure, lipid A, MCR-1, NDM-5, NDM-1, multi-drug resistance (MDR), colistin resistance, resistance to carbapenems

Antimicrobial resistance (AMR) is a big challenge threatening public health worldwide. Every year, AMR-associated bacterial infections result in over 70,000 people deaths in the United States [1,2]. Worryingly, it is estimated that AMR will claim for 10,000,000 lives globally per year by 2050 [3]. The major reasons for this macabre scenario are i) The rapid appearance of new AMR determinants; ii) The global transmission of epidemic pathogens with multi-drug resistance (MDR) or even extensive-drug resistance (XDR); and iii) The failure to invent new antimicrobial agents. In general, carbapenems are used as a last line of defense antibiotics against severe infections with MDR-possessing Enteriobactericeae. Colistin, a cationic antimicrobial polypeptide, is clinically regarded as a final line of defense to treat lethal challenges with carbapenem-resistant pathogens [4,5]. Unfortunately, it seems likely that the clinical use of carbapenems and colistin has been significantly challenged by the occurrence of mobilized colistin resistance determinant (MCR-1) in clinical carbapenem-resistant isolates producing New Delhi metallo-*β*-lactamase 1 (NDM-1) [6,7] or its variant NDM-5 [8,9]. In addition to the intrinsic determinants of colistin resistance (e.g., EptA [10,11] and ICR-Mo [12]), the transferable determinants (MCR-1 and MCR-2 [10,13,14]) consistently encode members of phosphoethanolamine (PEA)-lipid A transferases, which adopt a ‘ping-pong’ catalysis reaction in transferring of the PEA moiety to the 4ʹ-phosphate position of the lipopolysaccharide (LPS)-lipid A species anchored on bacterial surface [4,5].

In the two years since its first discovery in Southern China, in late of 2015 [15], *mcr-1* has been detected to coexist with *bla*_NDM-1_-like genes in migratory birds (like the Muscovy duck [16] plus chickens [17]), in healthy people [18] and also in patients with bloodstream infections [6,7,9,19]. This indicates that the possible route of *mcr-1*/*bla*_NDM_-like genes is through the food-chain transmission. With the exception of *Cronobacter sakazakii* that carries NDM-9 [20], *E. coli* seems to be the predominant reservoir for the coexistence of MCR-1 and NDM-like enzymes [6–9,16–19,21,22]. Surprisingly, *mcr-3*, a new *mcr*-like gene, was found in an XDR-*E. coli* clinical isolate which harbored both *mcr-1* and *bla*_NDM-5_ [23]. This might further highlight the importance of ‘one health’ approach in active surveillance of *mcr*-like genes in human, animal and environmental sectors. However, current knowledge on how MDR-bacterial pathogens emerge and evolve is relatively limited.

Here we report that antibiotic exposure elicits the emergence of a carbapenem- and colistin-resistant *E. coli* clinical isolate coharboring MCR-1 and NDM-5 in a hospitalized patient. On 3 June 2017, a 58-year-old man was admitted to the Lishui People’s Hospital of Zhejiang Province, China (Figure 1(A)), and was diagnosed to be suffering from Small Intestinal Perforation with Peritonitis (SIPWP). A routine examination of this patient suggested that neither immune-suppression nor specific comorbidity were present (Table S1). While in hospital, the inflammatory markers (temperature and Leukocyte count) of this patient were normal (Figure 1(B)). The patient was subjected to anti-infection therapy through 0.5 h of intravenous guttae containing 2.0g q6h of Piperacillin/Tazobactam (TZP) prior to surgical operation. To probe the suspected bacterial agent, the specimen of peritoneal fluid from the patient (Figure 1(A)) was inoculated on a petri plate with MacConkey agar. Antibiotic susceptibility assays revealed that all of the acquired *E. coli* isolates (designated as S-*E. coli*) are sensitive to most of the antibiotics including TZP (Table S2).

**Figure 1.**
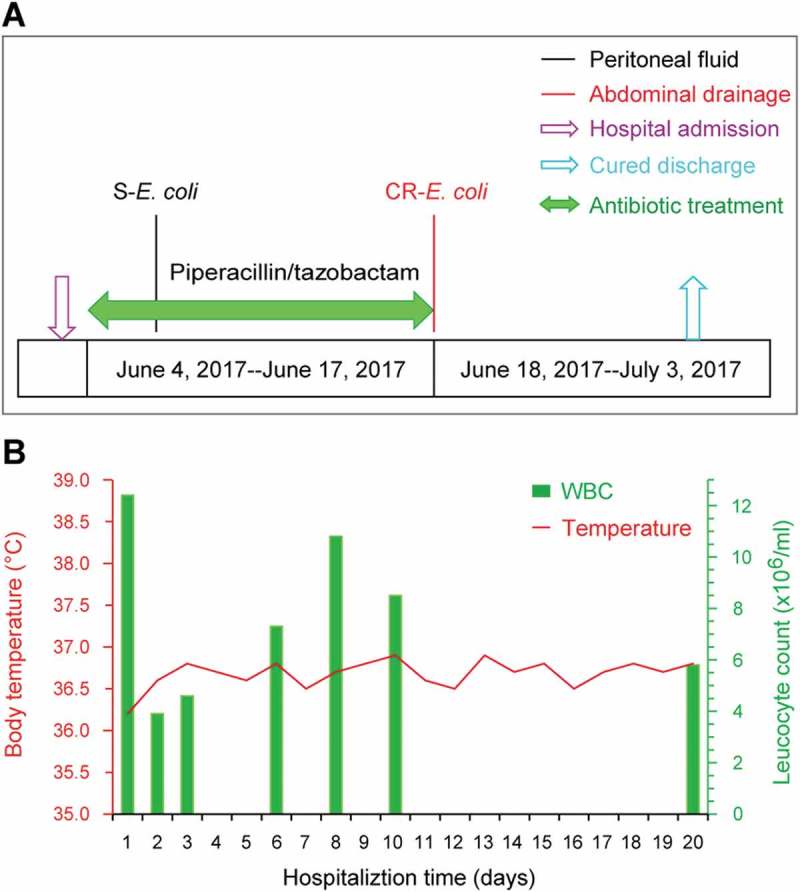
Clinical description of a patient undergoing antibiotic treatment. A. The history of antibiotic exposure for a patient admitted into the hospitalThe colored text and bars represent the sources from which the bacterial species were isolated. S-*E. coli*, sensitive*-E. coli*; CR-*E. coli*, Carbapenem-resistant *E. coli* B. Dynamic alterations of body temperature and leukocyte counts of the patient recorded during the hospitalization period

Following 2-weeks of antibiotic therapy, which proved to be a clinically-successful anti-infection treatment (Figure 1(A)), the abdominal drainage and the blood of the patient were sampled for bacterial cultivation. Although there were no *E. coli* isolates recovered from the patient’s blood samples during the entire treatment period, a MDR-*E. coli* isolate was unexpectedly detected in the abdominal drainage sample (Table S2). This purulent culture was not only found to be highly-resistant to imipenem and meropenem, two widely-used carbapenems, but also it displayed an appreciable level of resistance to colistin (Table S2). Thus, it was appropriately designated as CR-*E. coli* LSB54 (Figure 1(A)). In addition, we favored to speculate that the LSB54 strain is a representative of a small sub-population of local microbiota on this hospitalized patient, and it was elicited by an over-exposure to antibiotics. After the evaluation of the patient’s inflammatory index and the infective wound, the antibiotic treatment was immediately discontinued, but surgical debridement and drainage were resumed. As a result, the patient received a suitable course of treatment and was discharged after being hospitalized for 30 days.

To further address the purulent culture (CR-*E. coli* LSB54), we integrated multiple approaches ranging from microbial genetics, molecular biology, to comparative genomics. The diversity of genetic backgrounds was observed among the antibiotic-susceptible clinical strains from patients with similar durations of hospitalization (Table S3). In contrast, sequence typing showed that CR-*E. coli* belongs to ST2179 (Table S3). This data seems to rule out the possibility that the MDR-*E. coli* was acquired in the hospital. Of note, a ST2179 *E. coli* isolate with multi-drug resistance was recently found to claim for extra-intestinal infections in horses in Brazil [24]. Therefore, it seems likely that a possible transmission route for the zoonotic agent ST2179 is present between animals and humans. As we recently described with *mcr-1*-bearing plasmids carried by commensal E. coli [25], whole genome sequencing was performed, giving evidence that two unique plasmids coexist in the MDR-*E. coli* strain LSB54 (Figure 2–4). Among the two plasmids, pLSB54-*mcr-1* is the *mcr-1*-harboring plasmid (Figures 2 and 4(A)), and pLSB54-NDM-5 is the NDM-5-carrying plasmid (Figures 3 and 4(A)). Subsequent plasmid replicon typing (https://cge.cbs.dtu.dk/services/PlasmidFinder/) revealed that pLSB54-*mcr-1* (Acc. no.: MG773376) is a IncHI2-type plasmid with a genome size of 251.657 kb (Figure 2), whereas pLSB54-NDM-5 (Acc. no.: MG773377) is a IncX3-like plasmid whose genome is 46.19 kb long (Figure 3).

**Figure 2.**
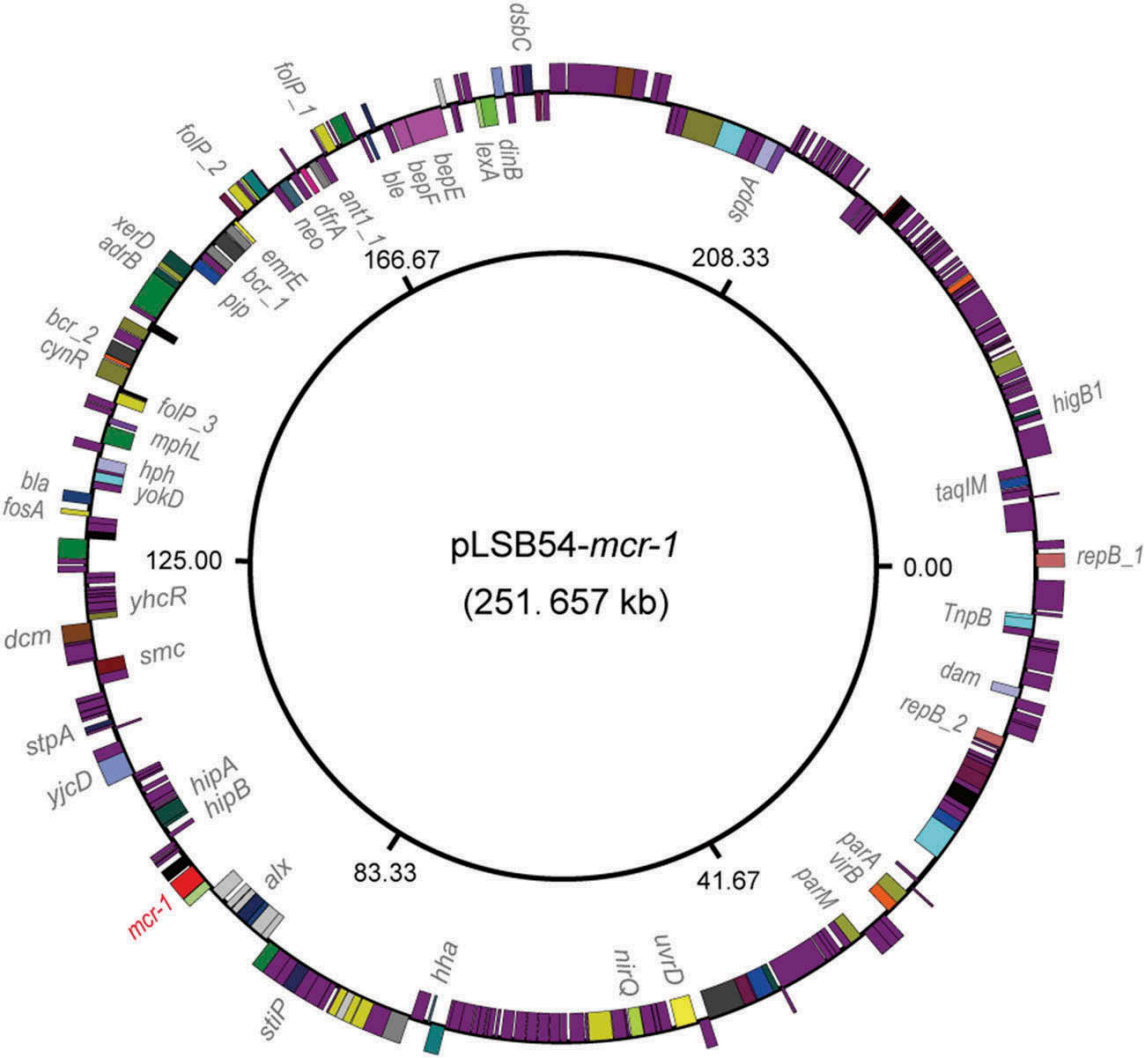
Genomic map of the *mcr-1*-carrying plasmid pLSB54-*mcr-1.* The plasmid map was generated using GenomeVx [28] with automatic coloring.

**Figure 3.**
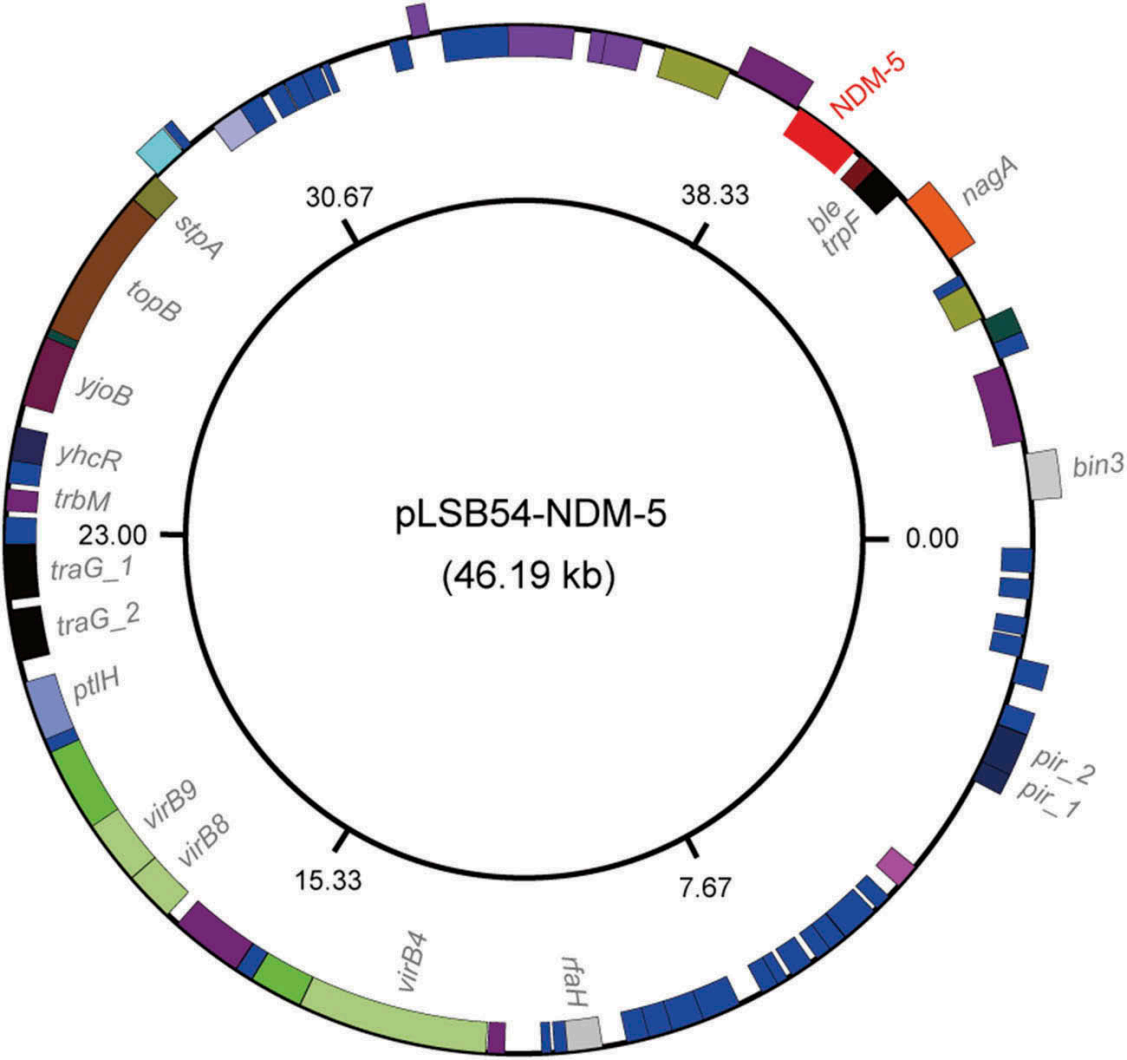
Genomic map of the NDM-5-producing plasmid pLSB54-NDM-5. The plasmid map was generated using GenomeVx [28] with automatic coloring.

**Figure 4.**
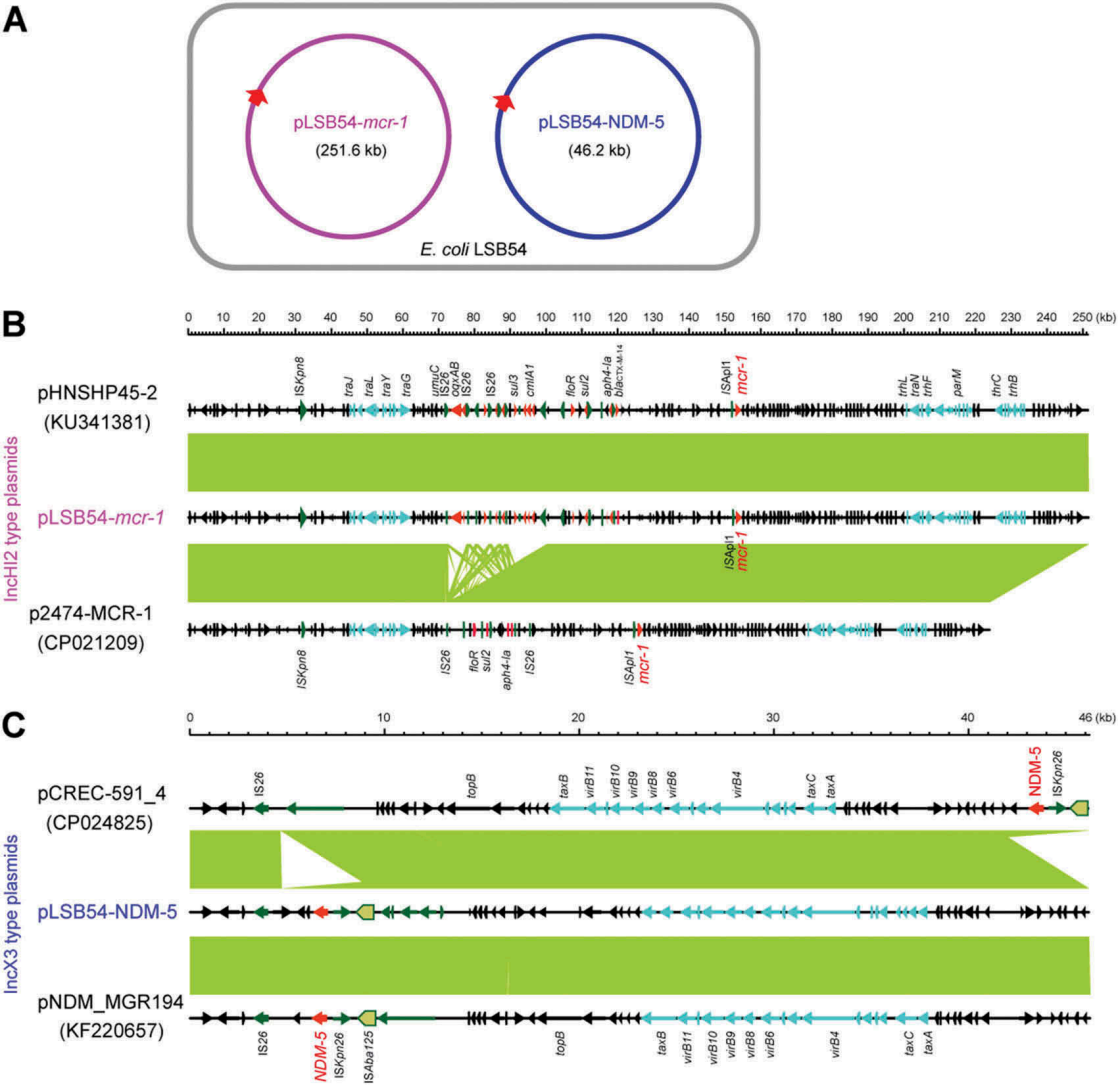
Comparative genomics of pLSB54-*mcr-1* and pLSB54-NDM-5. A. Cartoon description of the *E. coli* LSB54 strain carrying the two plasmids pLSB54-*mcr-1* and pLSB54-NDM-5B. Colinear genome analyzes of three *mcr-1*-harboring plasmids pLSB54-*mcr-1*, pHNSHP45-2 (KU341381) and p2474-MCR1 (CP021209)C. Genomic comparison of the plasmid pLSB54-NDM-5 with two other NDM-5-expressing plasmids pCREC-591_4 (CP024825) and pNDM_MGR194 (KF220657)Boxed arrows represent the position and transcriptional direction of the ORFs. Regions of > 99% identity are given by green shading. Genes associated with the *tra* and *pil* loci are colored light blue, antibiotic resistance genes are colored red, insertion sequences are colored green, and other genes are colored black.

Colinear alignment analyzes of the plasmid genomes indicated that pLSB54-*mcr-1* is almost identical with the prototypical *mcr-1*-harboring plasmid pHNSHP45-2 (Acc. no.: KU341381) (Figure 4(B)), and possesses 90% coverage and 99% identity with p2474-MCR1 (Acc. no.: CP021209) [26], another *mcr-1*-positive plasmid from a clinical *E. coli* isolate in China (Figure 4(B)) [6]. Given that pHNSHP45-2 is isolated from *E. coli* with origin of swine feces, it raised the possibility that origin of pLSB54-*mcr-1* might lie in animal gut microbiota. Intriguingly, p2474-MCR1 was also detected to coexist with the *bla*_NDM-1_-carrying plasmid in a single isolate of *E. coli* [6]. Like most of the known IncHI2-type plasmids, pLSB54-*mcr-1* is also positive for other antibiotic resistance genes (Table S4). Evidently, this is consistent with the phenotype of being resistant to multiple antibiotics which was observed with the strain LSB54 (Table S2).

The plasmid pLSB54-*mcr-1* encodes the following genes conferring resistance to aminoglycosides, β-lactam (*bla*_CTX-M-14_), fluoroquinolones (*oqxA* and *oqxB*), fosfomycin (*fosA3*), phenicols (*floR* and *cmlA1*), sulphonamides (*sul1, sul2* and *sul3*) and trimethoprim (*dfr12*) (Tables S2 and S4). As for pLSB54-NDM-5, it presents 100% coverage with the NDM-5-producing IncX3 plasmid pCREC-591_4 (Acc. no.: CP024825) of *E. coli* isolated from South Korea (Figure 4(C)) and exhibits a 99% identity to the plasmid pNDM_MGR194 (KF220657) of *Klebsiella pneumoniae* seen in India (Figure 4(C)). Whereas, the insertion loci of NDM-5 are quite different in the above three plasmids (Figure 4(C)). Unlike pLSB54-*mcr-1*, pLSB54-NDM-5 only contains a single resistance gene *bla*_NDM-5_ (Table S4). Because of the plasmid pNDM_MGR194 from human urine, we favored to anticipate that pLSB54-NDM-5 might originate from human microbiota.

Fine mapping of the genetic environment surrounding the *mcr-1*(*bla*NDM-5) gene further illustrated that i) In the IncHI2-type plasmid pLSB54-*mcr-1*, the insertion sequence IS*Apl1* is located upstream of the *mcr-1* gene (Figure 5(A)) and the IS*Apl1-mcr-1-pap2* fragment is inserted downstream of the *terF* gene, just like the plasmids pHNSHP45-2 (Acc. no.: KU341381) and p2474-MCR (Acc. no.: CP021209) (Figure 5(A)); ii) The NDM-5-containing fragment is identical in all three IncX3-like plasmids, including pLSB54-NDM-5 (Figure 5(B)), and the *bla*_NDM-5_ is adjacent to the insertion sequence IS*5*, which is flanked by a truncated IS*Aba125* sequence (Figure 5(B)). Unlike the scenario where MCR-1 and NDM-5 is co-transferred by a single hybrid IncX3-X4 plasmid in *E. coli* [8], *mcr-1* and *bla*_NDM-5_ are separately encoded by two distinct plasmids in the clinical *E. coli* isolate LSB54 (Figure 4(A)). In this clinical case, the MDR-*E. coli* which possesses resistance to colistin and carbapenem (two lines of last-resort antibiotics), emerges in a patient undergoing antibiotic treatment with TZP. This observation somewhat undermines rationality of anti-infection therapies (via inappropriate usage and/or overuse of antibiotics) to some extent. Very recently, we observed an increase in the antimicrobial activity of colistin against *E. coli* co-producing NDM-5 and MCR-1, when it was supplied with amikacin, which could be a promising therapeutic option for the treatment of lethal infections caused by MDR-bacterial pathogens [21].

**Figure 5.**
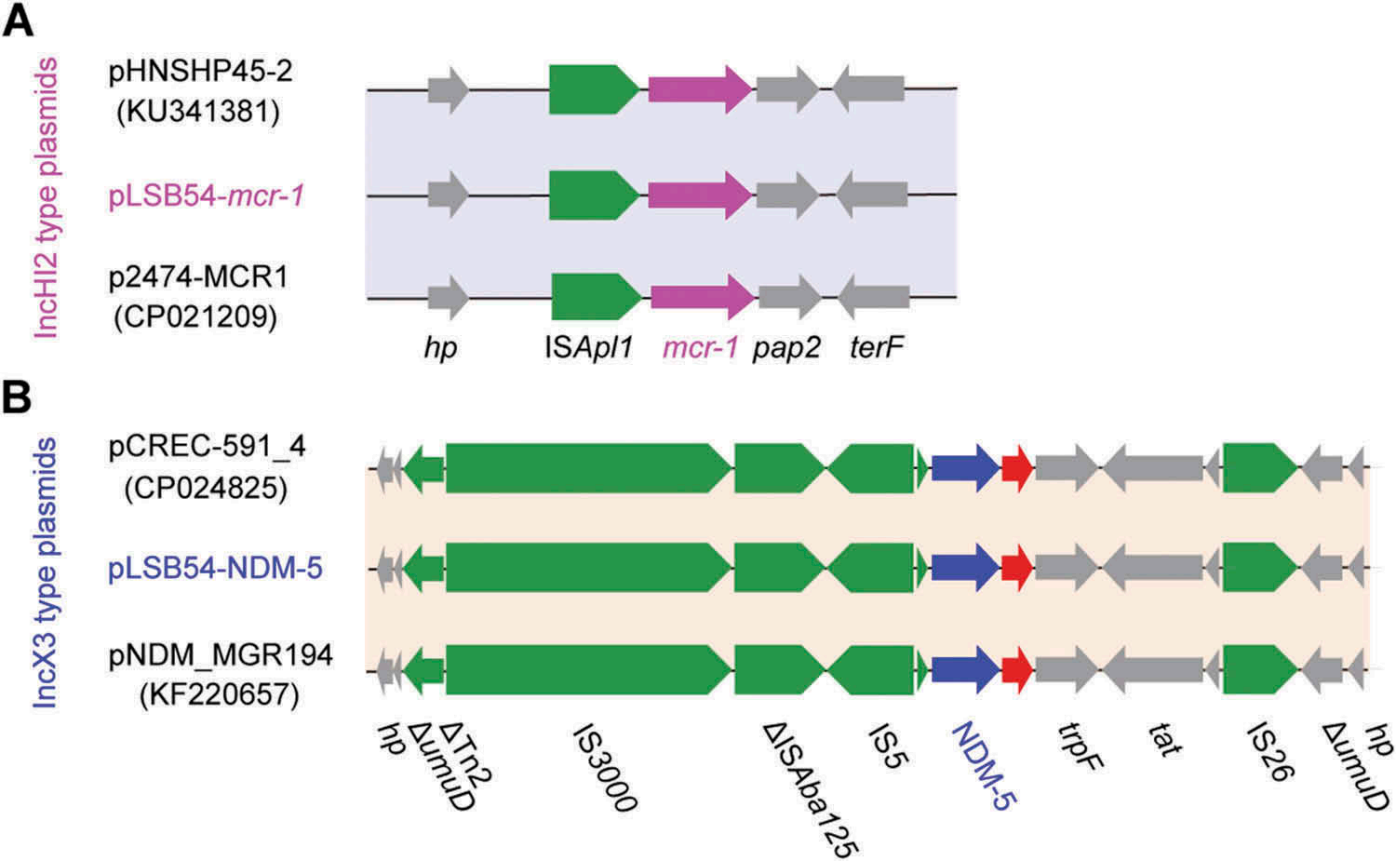
Genetic contents of *mcr-1* (and/or *bla*_NDM-5_). A. The genetic environment of *mcr-1* and its neighboring lociB. The genetic environment of *bla*_NDM-5_ and its neighboring lociThe arrows indicate open reading frames, resistance genes, insertion sequences and accessory genes are separately shown with red, green and gray arrows.

In summary, our findings report the first clinical case that antibiotic exposure elicited the emergence of MDR-*E. coli* coharboring MCR-1 and NDM-5 in a patient. The fact that the co-existence of MCR-1 and NDM variants has been found in bacterial species isolated from humans, animals and environments [6,8,16,19,27], reminded us to employ the approach of ‘one health’ in combating potential challenges with MDR-pathogens. Given that inappropriate use of antibiotics is a potential threat for the rapid development of drug resistance and therapeutic failure, it is prerequisite to reconsider (and/or screen) the subpopulations of MDR-pathogens persisting in human microbiota prior to the formulation of appropriate antibiotic-based effective anti-infection therapies.

## Availability of data and materials

All the data are included in this manuscript. Complete sequences of the two plasmids were deposited into GenBank under the accession no. (MG773376 for pLSB54-*mcr-1*, MG773377 for pLSB54-NDM-5).
