# Supplementary material for: Antibiotic exposure elicits the emergence of colistin- and carbapenem-resistant Escherichia coli coharboring MCR-1 and NDM-5 in a patient
Source: Virulence. 2018 Jul 26;9(1):1001–7. doi: 10.1080/21505594.2018.1486140 (PMC6067848; doi:10.1080/21505594.2018.1486140)
Supplement: Supplemental Material [file kvir-09-01-1486140-s001.doc]

**Supplementary Materials for**

**Antibiotic exposure elicits the emergence of colistin- and carbapenem -resistant *Escherichia coli* coharboring MCR-1 and NDM-5 in a patient**

Jinpeng Mao1#, Wugao Liu2#, Wei Wang2#, Jian Sun3, Sheng Lei4, Youjun Feng3,4,5*

This SOM includes 4 supplementary tables (Table S1, Table S2, Table S3, and Table S4), Supplementary Methods, Supplementary References.

**Supplementary Tables**

**Table S1** Clinical characteristics and outcomes of a hospitalized patient

| Age, Sex | 58, M |
| --- | --- |
| Specific Comorbidities | no |
| Immuno-suppression status | no |
| Community-acquired infections | Yes |
| Post-surgical infections | No |
| Acute physiology and chronic health evaluation | 15 |
| Fever/Hypotension | No/Yes |
| Microbiologic characteristics | CRE |
| Culture source | peritoneal fluid |
| Polymicrobial culture | CRE |
| Meropenem susceptibility | R (>16) |
| Management | |
| Source control achieved (surgery) | Yes |
| Time prior to effective antibiotic therapy (hs) | 72 |
| Duration of effective antibiotic therapy (days) | 14 |
| Outcomes | |
| Length of hospital stay (days) | 30 |
| In-hospital death | No (Survived) |
| Relapse | No |
| Death attributable to infection | No |

**Table S2** Antibiotic susceptibility assays of the *E. coli* clinical isolates

| Antibiotics | S-*E. coli* | | CR-*E. coli* | |
| --- | --- | --- | --- | --- |
|  | MIC/Antibiotic susceptibility | | | |
| **CL** | **≤0.5** | **S** | **8** | **R** |
| ATM | ≤1 | S | ≥64 | R |
| ETP | ≤0.5 | S | ≥6 | R |
| **IPM** | **≤1** | **S** | **≥16** | **R** |
| AK | ≤2 | S | ≤2 | S |
| GN | ≤1 | S | ≥16 | R |
| TOB | ≤1 | S | ≥16 | R |
| CIP | ≤0.25 | S | ≥4 | R |
| LEV | ≤1 | S | ≥8 | R |
| SXT | ≥320 | R | ≥320 | R |
| AMP | ≥32 | R | ≥32 | R |
| **MEM** | **≤0.5** | **S** | **≥16** | **R** |
| SAM | 16 | I | ≥32 | R |
| TZP | ≤4 | S | 64 | R |
| KZ | ≤4 | S | ≥64 | R |
| CTT | ≤4 | I | ≥64 | R |
| CAZ | ≤1 | S | ≥64 | R |
| CRO | ≤1 | S | ≥64 | R |
| FEP | ≤1 | S | 16 | R |

CL, Colistin; ATM, Aztreonam; ETP, Ertapenem; IPM, Imipenem; AK, Amikacin; GN, Gentamicin; TOB, Tobramycin; CIP, Ciprofloxacin; LEV, Levofloxacin; SXT, Trimethoprim/Sulfamethoxazole; AMP, Ampicillin; MEM, Meropenem; SAM, Ampicillin/Sulbactam; TZP, Piperacillin/Tazobactam; KZ, Cefazolin; CTT, Cefotetan; CAZ, Ceftazidime; CRO, Ceftriaoxone; FEP, Cetepime

MIC, minimum inhibitory concentration

**Table S3** Sequence typing of *E. coli* strains

| Strains* | Allelic loci | | | | | | | ST | Origins |
| --- | --- | --- | --- | --- | --- | --- | --- | --- | --- |
|  | *adK* | *fumC* | *gyrB* | *icd* | *mdh* | *purA* | *recA* |  |  |
| **­­LSB54** | 9 | 65 | 5 | 18 | 11 | 8 | 6 | **ST2179** | Pus |
| K240 | 10 | 11 | 4 | 8 | 8 | 8 | 2 | ST10 | Urine |
| K241 | 6 | 19 | 33 | 26 | 11 | 8 | 6 | ST602 | Blood |
| K242 | 6 | 29 | 14 | 18 | 11 | 8 | 14 | ST4657 | Blood |
| K243 | 37 | 38 | 19 | 37 | 17 | 18 | 26 | New | Umbilical cord blood |
| K244 | 6 | 4 | 33 | 16 | 11 | 8 | 6 | ST224 | Blood |
| K245 | 53 | 40 | 47 | 13 | 36 | 28 | 29 | ST131 | Urine |
| K246 | 10 | 11 | 4 | 8 | 8 | 8 | 2 | ST10 | Urine |
| K247 | 6 | 4 | 33 | 16 | 11 | 8 | 6 | ST224 | Urine |
| K248 | 37 | 38 | 19 | 37 | 17 | 11 | 26 | ST95 | Blood |
| K249 | 14 | 14 | 10 | 200 | 17 | 7 | 10 | ST1193 | Umbilical cord blood |

*The 10 clinical *E. coli* strains (K240-K249) that are sensitive to most of tested antibiotics were collected from different patients with almost similar durations of hospitalization.

**Table S4** Antimicrobial resistance genes identified from the two plasmids pLSB54-*mcr-1* and pLSB54-NDM-5

| Plasmids | Antimicrobial resistance genes |
| --- | --- |
| pLSB54-*mcr-1* | *aph*(4)*-Ia, aac*(3)*-Iva, aadA2, aph*(3')*-Ia, aadA1, aadA2, bla*_CTX-M-14_*, mcr-1, oqxA, oqxB, fosA3, floR, cmlA1, sul1, sul2, sul3, dfrA12* |
| pLSB54-NDM-5 | *bla*_NDM-5_ |

*aph*(4)*-Ia,* a hygromycin B resistance; *aac*(3)*-Iva,* gentamicin resistance; *aph*(*3'*)*-Ia*, Aminoglycoside resistance; *aadA1/aadA2*, Aminoglycoside resistance; *bla*_CTX-M-14_, β-lactam resistance; *mcr-1*, Colistin resistance; *oqxA*, Quinolone resistance; *oqxB*, Quinolone resistance; *fosA3*, Fosfomycin resistance; *floR*, Phenicol resistance; *cmlA1*, Phenicol resistance; *sul1/sul2/sul3*, Sulphonamide resistance; *dfrA12*, Trimethoprim resistance; *bla*_NDM-5_, Carbapenem resistance

**Supplementary Methods**

**Clinical data and ethics statement**

The antimicrobial therapeutics of the inpatient was conducted in Lishui People’s Hospital. All the clinical data was collected by Dr. W Liu, which is according to the guideline of the local ethics committee from the Lishui People’s Hospital (2017-005-01).

**Microbial and molecular analyses**

Purulent cultures from the inpatient were examined on a petri plate with MacConkey agar. Multiplex-PCR with specific primers (like 16S rDNA) was conducted to determine bacterial identity. To figure out the diversity of *E. coli* population, multiple loci sequence typing was utilized. As for the isolated *E. coli* clinical isolates, antibiotic susceptibility assays (such as colistin and meropenem) were carried out.

**Genomic sequencing and analyses**

The plasmids isolated from clinical *E. coli* were subjected to whole genome sequencing using the Illumina HiSeq X-ten platform (Illumina, USA). The resultant 150 bp paired-end reads were assembled with the software of SPAdes [version 3.11.0] into a series of contigs. Of note, the gaps in the *mcr-1* (and/or *ndm-5*)-containing contigs were closed with PCR and sanger sequencing. Plasmid replicon types were assayed with the program of PlasmidFinder 1.3 (<https://cge.cbs.dtu.dk/services/PlasmidFinder/>). Functional assignment of genes in the plasmids was performed with the RAST software, and the plasmid maps were created using GenomeVx .

**Nucleotide sequence accession numbers**

The two plasmids we reported here refers to pLSB54-*mcr-1* and pLSB54-NDM-5, respectively. The complete sequences of the two plasmids were separately deposited into GenBank under the accession no. (MG773376 for pLSB54-*mcr-1*, MG773377 for pLSB54-NDM-5).

**Supplementary References**

1. Conant GC, Wolfe K**H**. 2008. GenomeVx: simple web-based creation of editable circular chromosome maps. Bioinformatics 24:861-2.
